# BCOR::CCNB3 Sarcoma of the Thigh: A Rare Case

**DOI:** 10.7759/cureus.89654

**Published:** 2025-08-08

**Authors:** Abdul Moiz Khan, Saliq Naeem, Amna Ehtisham, Rehan Ali, Hamais Murtaza

**Affiliations:** 1 Internal Medicine, Sahiwal Medical College, Sahiwal, PAK; 2 General Medicine, Sahiwal Medical College, Sahiwal, PAK

**Keywords:** bcor-ccnb3, clinical pathology, ewing-like sarcoma, immunohistochemistry(ihc), sarcoma soft tissue

## Abstract

BCOR-rearranged sarcoma is a rare mesenchymal tumor and a recognized subtype of undifferentiated small round-cell sarcoma. It shares morphological similarities with other round-cell sarcomas but is distinguished by a unique molecular hallmark that differentiates it from Ewing sarcoma. These tumors primarily arise in bones and soft tissues. This case report details the case of an 11-year-old female who developed swelling on the medial aspect of her right distal thigh. There was no history of trauma, surgery, or fracture. Clinically, the presentation initially suggested osteosarcoma. However, histopathological evaluation confirmed a diagnosis of BCOR-rearranged sarcoma. This case highlights the importance of considering BCOR-rearranged sarcoma in the differential diagnosis of pediatric bone and soft tissue tumors, as its clinical and radiological features can mimic more common malignancies like osteosarcoma. Early recognition and accurate molecular diagnosis are crucial for guiding appropriate treatment.

## Introduction

Undifferentiated small round-cell sarcomas are rare, high-grade mesenchymal tumors composed of primitive small round cells, primarily involving bone and soft tissue. By definition, they lack specific differentiation or characteristic markers, resulting in nonspecific histology that overlaps with other round-cell neoplasms and thus poses significant diagnostic challenges. Ewing sarcoma is an undifferentiated small round cell sarcoma type having molecular markers as the translocation of a gene from the ETS family and EWSR1/FUS (a gene that encodes a protein that functions in gene expression, cell signaling, and RNA processing and transport) in some rare cases [1]. Similarly, Ewing-like sarcomas get their name for the similarities in morphology with a mass of small round cells but differences in genetic alterations from Ewing sarcoma [2]. Major subtypes of Ewing-like sarcomas include CIC-rearranged sarcomas, which are typically associated with a CIC-DUX4 fusion. These tumors tend to exhibit more aggressive behavior and are generally less responsive to conventional Ewing sarcoma therapies. Another subtype is BCOR-rearranged sarcomas, commonly involving a BCOR-CCNB3 fusion, and they predominantly arise in the bone and soft tissues of adolescents and young adults. BCOR-rearranged sarcoma is characterized by a specific in-frame fusion of the BCOR and CCNB3 genes, its hallmark molecular change. This fusion results from a hidden paracentric inversion on the X chromosome, linking the 3′ end of BCOR (Xp11) to CCNB3, forming a chimeric transcript that leads to abnormal BCOR/CCNB3 expression. Other rare variants exist, including NUTM1-rearranged sarcomas, each with distinct genetic profiles and clinical characteristics.

Since its discovery in 2012 through RNA sequencing of bone tumors, BCOR-rearranged sarcoma has become the third most common small round cell sarcoma subtype [1]. It may consist of rearrangements such as BCOR-CCNB3, BCOR-MAML3, and ZC3H7B-BCOR [3]. The most common rearrangement is BCOR-CCNB3 fusion, which results from paracentric inversion of the X-chromosome between exon 15 of BCOR and exon 5 of CCNB3 and serves as a key role in its oncogenic potential [1].

BCOR::CCNB3 sarcoma is a newly recognized and exceptionally uncommon variant of undifferentiated round cell sarcoma, representing less than 1% of all soft tissue sarcomas. With fewer than 150 cases documented in the literature, it remains a rare entity that poses significant diagnostic challenges. Primarily, BCOR-rearranged sarcomas were predominantly reported in bones and sporadically found in extraosseous tissues involving the kidney, neck, chest wall, and soft palate, with a preferred morbidity in male adolescents and children [1]. In extensive studies, patients' ages at diagnosis varied widely (e.g., 2-44 years, median ~15 years), with a notable male predominance (roughly 6:1 male-to-female ratio) [4]. The five-year overall survival (OS) rate for BCOR-CCNB3 sarcoma is moderate. A multicenter study reported a five-year OS of approximately 72%, similar to classical Ewing sarcoma (~79%) and significantly higher than CIC-DUX4 sarcoma (~43%) [4].

Nonetheless, there have been very few cases of primary BCOR-rearranged sarcoma in Pakistan, and because it mimics the morphology of Ewing sarcoma and osteosarcoma, the diagnosis and treatment of BCOR-rearranged sarcoma can be challenging. Therefore, an integrated approach is required for an accurate diagnosis and successful management of this condition. Here, we describe an adolescent female child with a rare diagnosis of BCOR-rearranged sarcoma. The condition was initially thought to be similar to Ewing sarcoma or osteosarcoma. This highlights the importance of histopathological and immunohistochemical correlation for an accurate diagnosis of Ewing-like sarcomas, as well as the need for molecular studies to confirm the diagnosis.

## Case presentation

**Figure 1:**
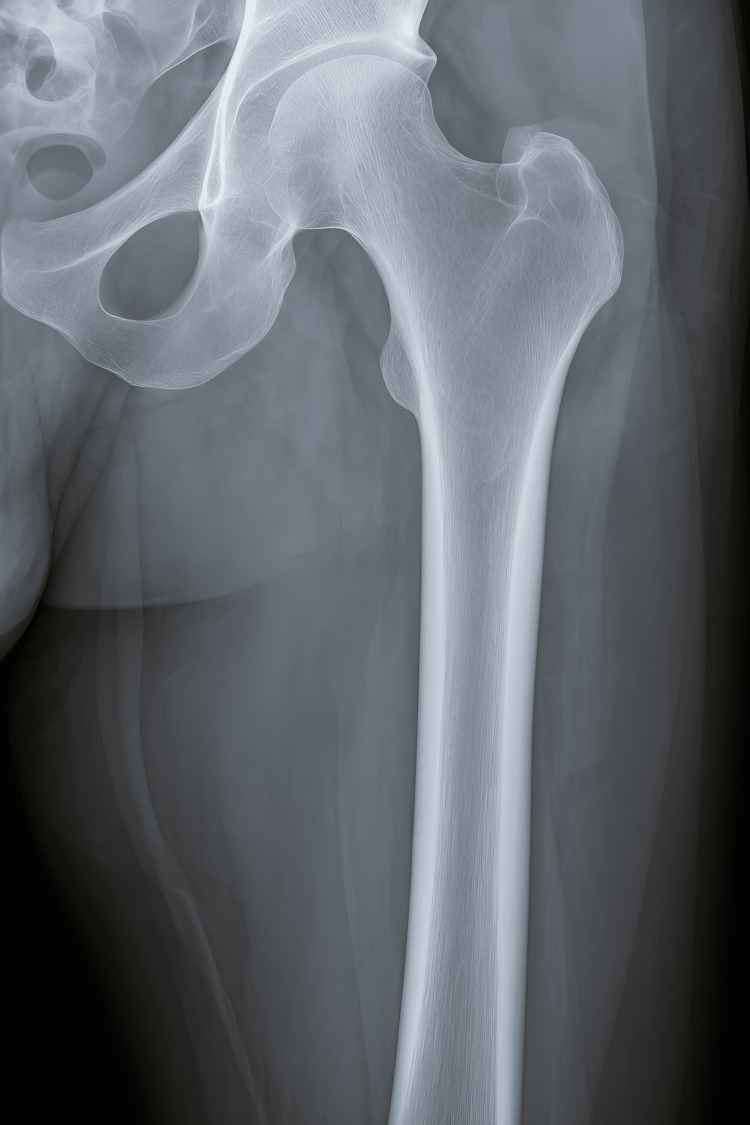
Partially circumscribed soft tissue swelling bulge is seen along the medial aspect of the left upper thigh. No soft tissue calcifications are seen.

An 11-year-old girl presented with a two-year history of knee pain and a slowly growing mass on the medial aspect of the left thigh with no notable cause. There was no concerning medical history, family history, or any other systemic symptoms like fever, vomiting, or nausea. Physical examination revealed a hard mass without tenderness, with mild warmth. A radiograph was taken, which showed an expansion on the left femoral shaft (Figure 1). She had no significant past history of trauma, fracture, or surgery to the lower limbs. Complete blood count was performed, which came out to be normal (Table 1). Urinalysis and liver function tests performed were also normal (Table 2).

**Table 1:** Liver function tests and urinalysis

Conventional system			International system	
Test name	Result	Reference range	Result	Reference range
Bilirubin total	0.590	0.3-1.2 mg/dL	10.1	5.1-20.5 µmol/L
Alanine aminotransferase	16.3	4-42 U/L	16.3	4-42 U/L
Creatinine	1.04	0.6-1.3 mg/dL	92.8	53-114.9 µmol/L
Sodium	136	136-146 mEq/L	136	136-146 mmol/L
Alkaline phosphate	76	40-130 U/L	76	40-130 U/L
Urea	20	13-43 mg/dL	3.3	2.2-7.1 mmol/L

**Table 2:** Complete blood count (CBC) table

Test	Result	Reference range
White cell count	6,000	4,000-10,000/µL
Red blood cell count	4.9	4.5-5.5 million/µL
Hemoglobin	13.9	13-17 g/dL
Mean corpuscular volume	87.2	80-98 fL
Hematocrit	42.9	40-50%
Platelet count	291,000	150,000-400,000/µL
Differential leukocyte count		
Neutrophils	65.3	45-70%
Lymphocytes	31.2	20-40%
Eosinophils	3.8	1-6%
Basophils	0.7	0.5-1%
C-reactive protein	10	<5

**Figure 2:**
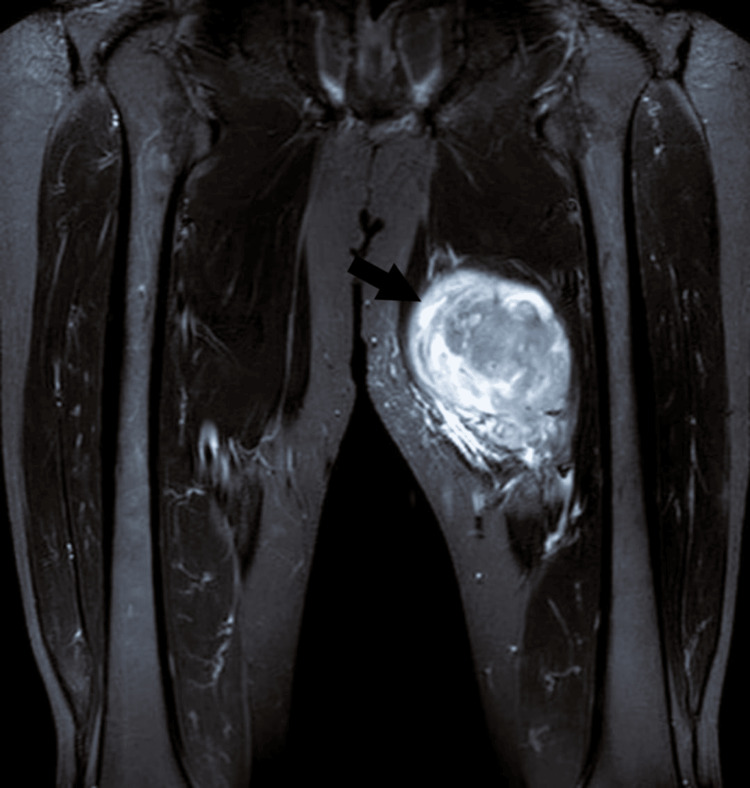
Axial T2-weighted MRI showing a hyperintense, well-defined mass in the thigh with surrounding edema.

The stool sample for microscopy, sensitivity, and culture did not reveal any infective etiology. Axial T2-weighted MRI performed showed a hyperintense, well-defined mass in the thigh with surrounding edema (Figure 2). Findings were suggestive of a soft tissue tumor or complex fluid-filled lesion. The coronal short tau inversion recovery (STIR) MRI showed a well-defined, hyperintense soft tissue mass in the left thigh, likely involving the adductor muscle group. The lesion's bright signal indicated a high water content. Coronal T1-weighted MRI showed a well-defined, isointense to slightly hypointense soft tissue mass in the left thigh. The mass appeared distinct from the surrounding muscle. 

**Figure 3:**
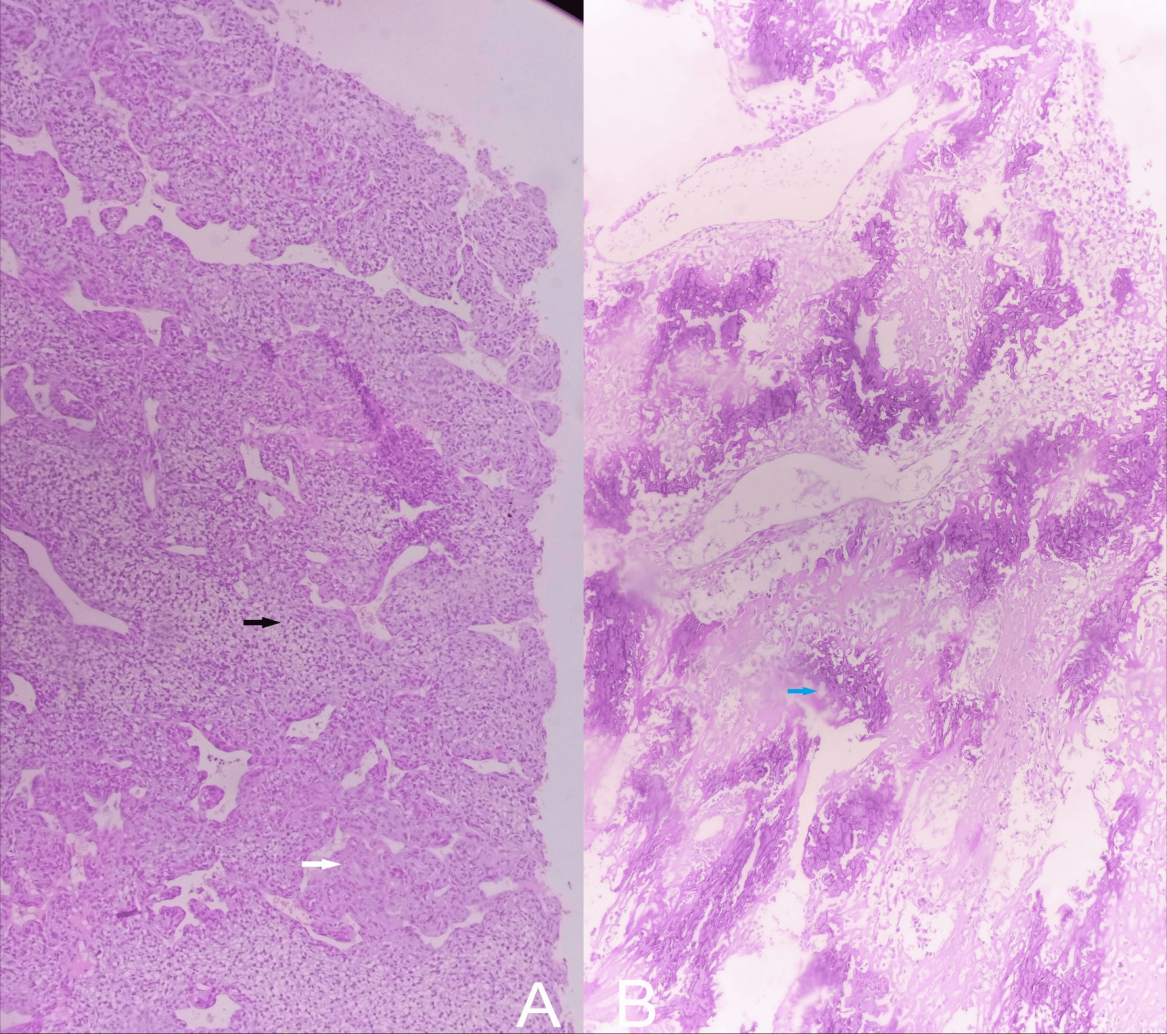
Microscopic images showing clusters of soft epithelioid to spindle cells (black arrow) arranged in nests and cords against a myxoid background (white arrow) (A) and scattered appreciable calcific flakes (blue arrow) (B).

**Figure 4:**
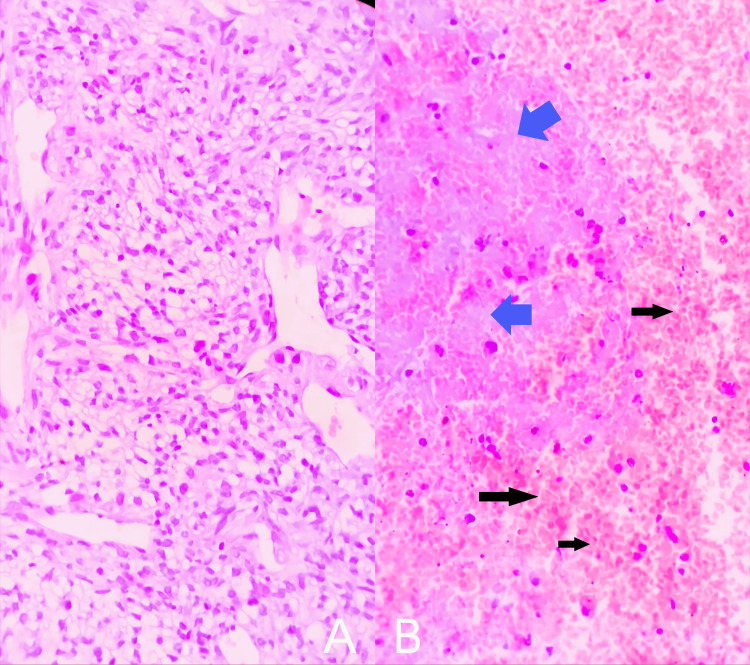
Microscopic images of the tumor showing focal areas of clear cell change seen in some sections (A) and areas of necrosis (blue arrows) and hemorrhages (black arrows) (B).

She visited a tertiary care setup where it was considered to be a soft tissue swelling, and on physical examination, it appeared to be an elastic-hard mass in her distal thigh, measuring about 6.4 × 5.5 × 4 cm. It was non-tender on palpation, and local inflammation with heat was prominent. A biopsy of the tumor revealed a high-grade soft tissue sarcoma, with a probable diagnosis of extraskeletal osteosarcoma, with a French Grading system for Soft Tissue Sarcomas (French Federation of Cancer Centers Sarcoma Group (FNCLCC)), Histological Grade 3 (Score 6). Microscopically, the tumor is composed of loose sheets of ovoid to spindle-shaped atypical cells demonstrating notable variation in cell and nuclear size and shape. The cells were arranged in nests and cords, set in a prominent myxoid background interspersed with dense collagenous stroma (Figure 3A). There were no foci of osteoid production characteristic of osteosarcoma, though there are scattered calcific flakes (Figure 3B), which support the impression of osteosarcoma. There are focal areas of clear cell change (Figure 4A), scattered necrosis, hemorrhages, and a high mitotic rate of 10-11 per 2 mm², indicating aggressive behavior (Figure 4B).

**Figure 5:**
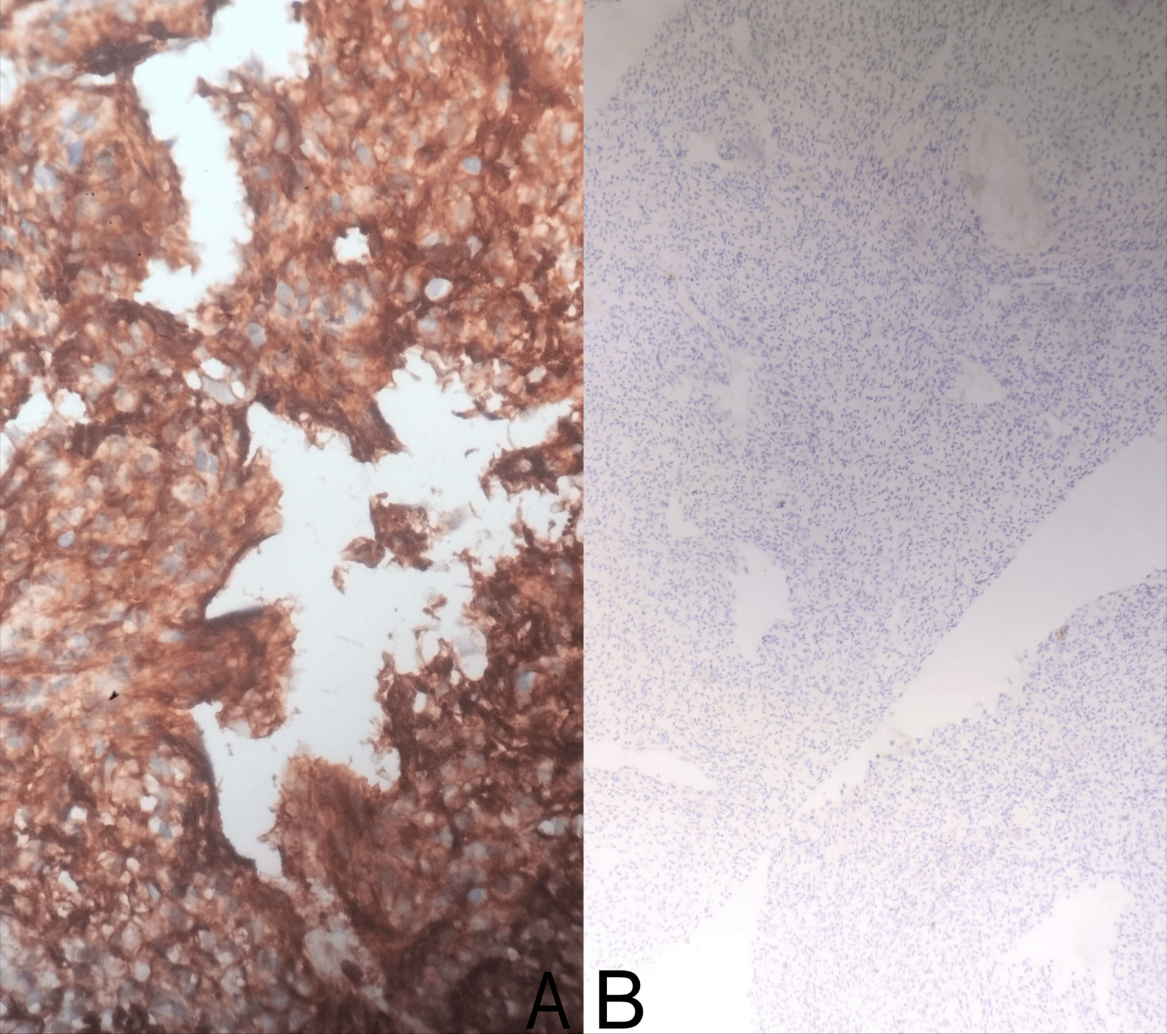
Microscopic images showing CD99 diffusely positive on immunohistochemistry, favoring Ewing and Ewing-like Sarcomas diagnosis (5A), and NKX2.2 negativity on immunohistochemical staining, ruling out Ewing sarcoma (5B).

**Figure 6:**
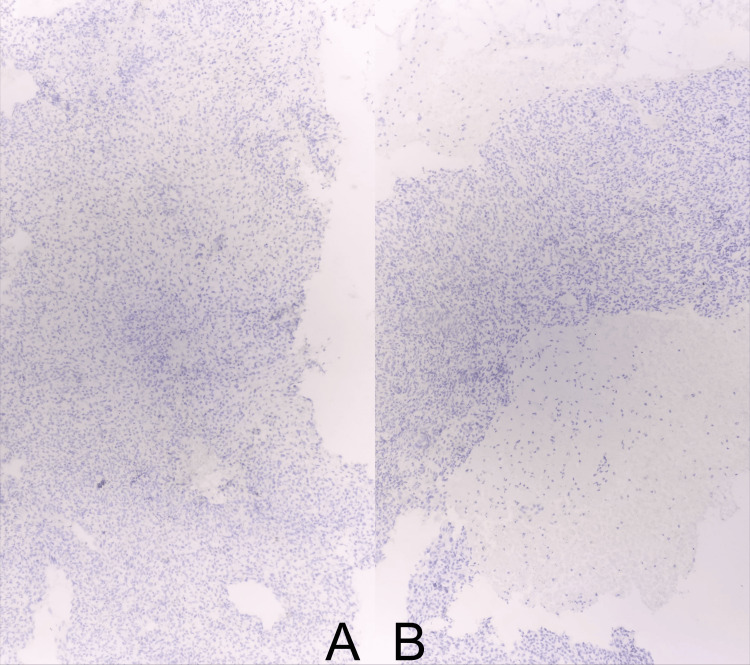
Microscopic images showing S-100 negativity (A) and HMB-45 negativity (B) on immunohistochemical staining.

**Figure 7:**
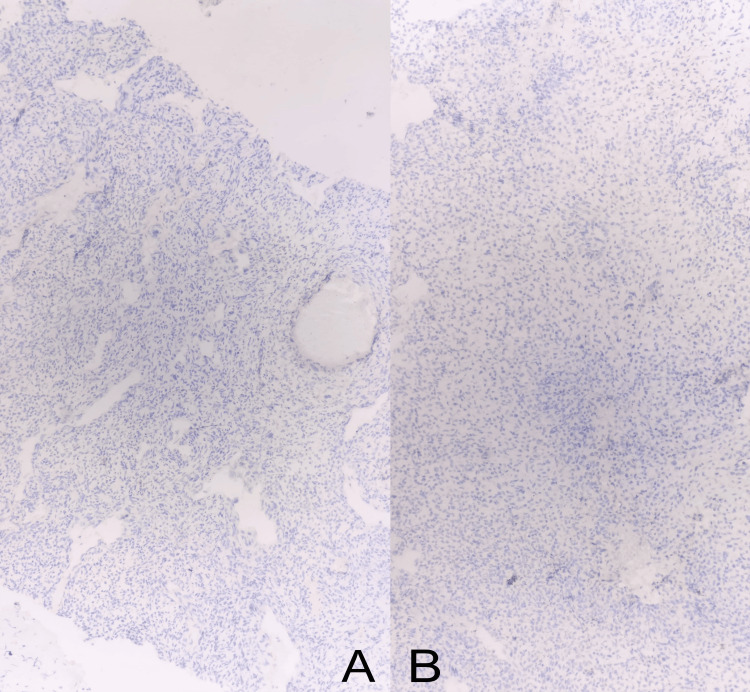
Microscopic images showing Desmin negativity on immunohistochemical stain, ruling out rhabdomyosarcoma (A), and PanCK negativity, ruling out synovial sarcoma (7B).

**Figure 8:**
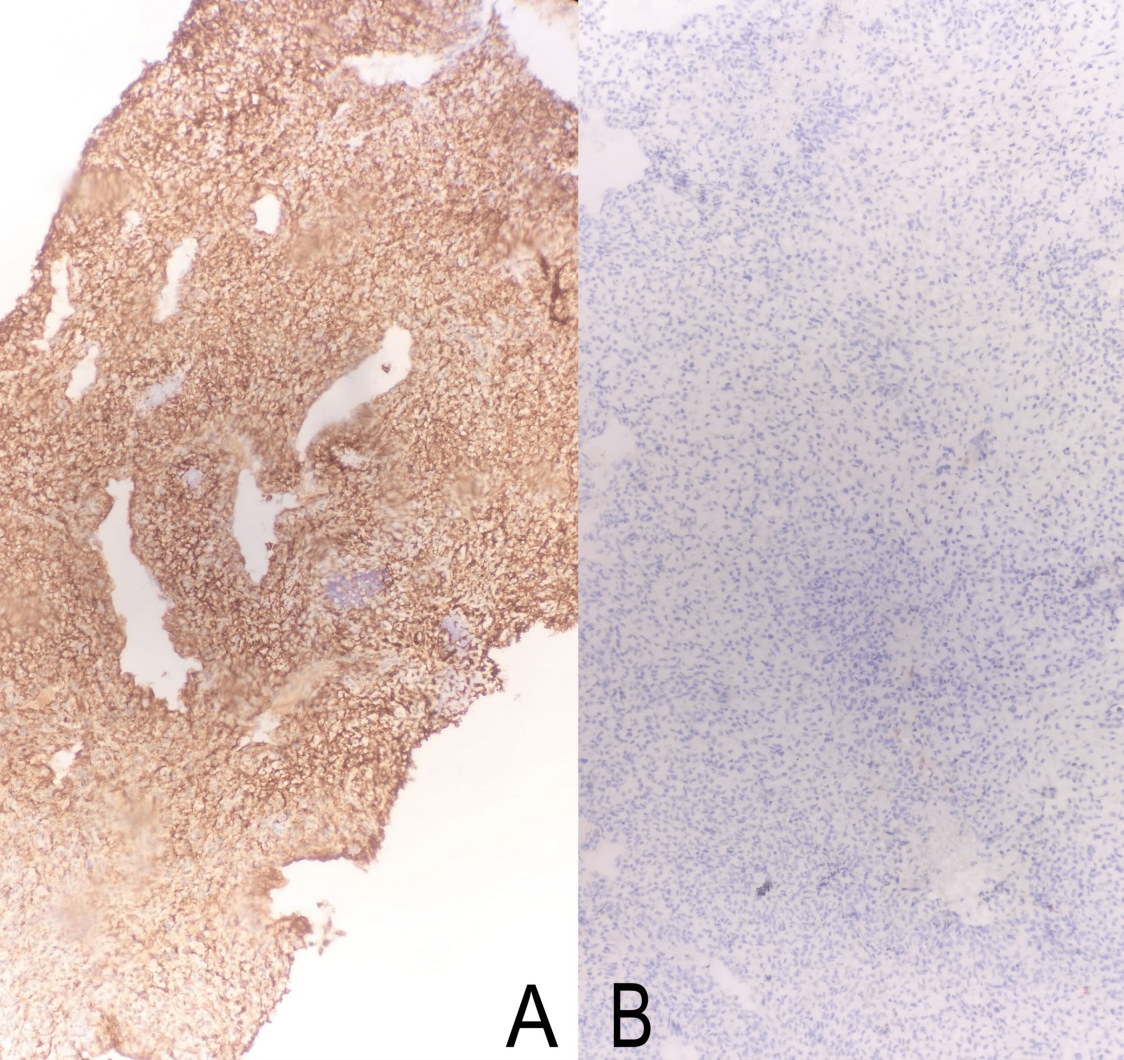
Microscopic images showing CD56 positivity on immunohistochemical stains, narrowing the differential to Ewing-like sarcomas, i.e., CIC-DUX4 and BCOR-CCNB3 sarcomas (A), and WT1 negativity, ruling out CIC-DUX4 Ewing-like sarcoma (B).

Further immunohistochemical staining was applied to rule out differential diagnoses of osteosarcoma, Ewing sarcoma, Ewing-like sarcoma (novel entities like CIC-DUX4 and BCOR-CCNB3 sarcomas), clear cell sarcoma of soft tissue, and other soft tissue tumors, e.g., rhabdomyosarcoma and synovial sarcoma. CD99 came positive, favoring a differential of Ewing sarcoma or Ewing-like sarcomas (Figure 5A). NKX2.2 negativity ruled out Ewing sarcoma as it is highly specific for Ewing sarcoma (Figure 5B). S-100 (Figure 6A) and HMB45 (Figure 6B) were negative, ruling out clear cell sarcomas of soft tissue. Desmin (Figure 7A) and PanCK (Figure 7B) came out negative, ruling out rhabdomyosarcoma and synovial sarcoma, respectively. CD56 came out positive (Figure 8A), narrowing the differential to Ewing-like sarcomas, i.e,. CIC-DUX4 and BCOR-CCNB3 sarcomas. WT1 came out negative, ruling out CIC-DUX4 Ewing-like Sarcoma (Figure 8B).

**Figure 9:**
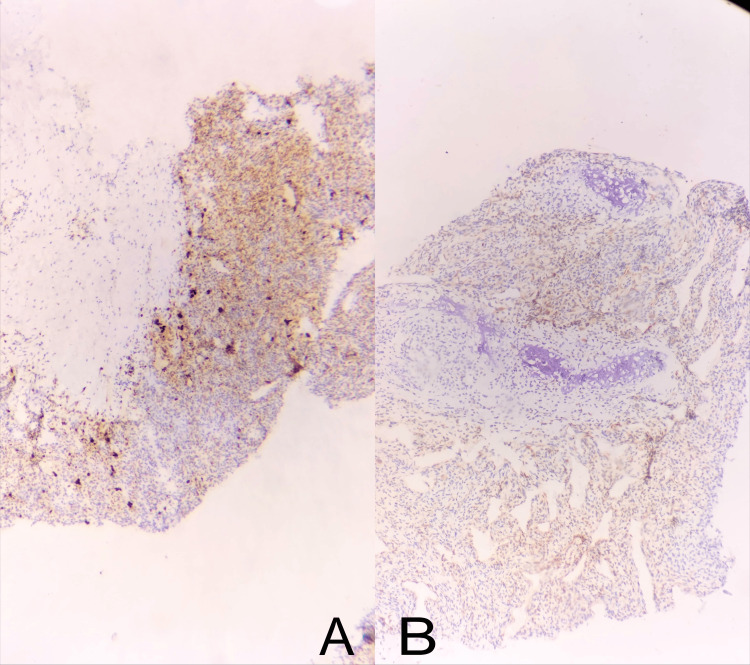
Microscopic images showing Cyclin D1 (A) and BCL2 (9B) positivity on immunohistochemical stain ruling in BCOR-CCNB3 sarcoma diagnosis.

CD99, CD56, Cyclin D1 (Figure 9A), and BCL2 (Figure 9B) came positive, whereas NKX2.2 was negative. Immunohistochemistry for CCNB3 was not used due to the unavailability of the marker. Therefore, further workup, including molecular studies, BCOR::CCNB3, was done for confirmation, which came out positive by fluorescence in situ hybridization (FISH) study. Furthermore, EWSR1 and SS18 gene rearrangement were not detected by the FISH study, confirming Ewing and Synovial sarcoma negativity. The patient was on three courses of alternative chemotherapy using vincristine, doxorubicin, cyclophosphamide, ifosfamide, and etoposide (VDC/IE), and as a result, the size of the mass started to decrease. The patient underwent monthly follow-up visits for two years and showed no signs of recurrence.

## Discussion

The current WHO Classification of Soft Tissue and Bone Tumours identifies four principal groups of undifferentiated small round cell sarcomas: Ewing sarcoma, round cell sarcomas with EWSR1-non-ETS fusions (such as NFATc2 and PATZ1), CIC-rearranged sarcomas, and sarcomas with BCOR genetic abnormalities [5]. The tumors are challenging to diagnose since they are rare and share overlapping morphological and immunohistochemical features; hence, it is essential to carry out molecular analysis for proper classification.

In studies of BCOR-CCNB3 sarcoma, patients are typically young males. A study by Kao et al. noted that patients' ages ranged from two to 44 years, with a median age of 15 [4]. The patient population was predominantly male, with 86% of cases occurring in males, a male-to-female ratio of 31:5. The tumors were more frequently found in bone (20 cases) compared to soft tissue (14 cases) [4]. Pierron et al. reported in 2012 that BCOR-CCNB3 sarcomas are a subgroup of Ewing-like sarcomas that have a common occurrence in bone and soft tissues [6]. BCOR-CCNB3 sarcoma was formally established as a distinct tumor in the 2020 WHO Classification of Soft Tissue and Bone Tumours. It belongs to the category of undifferentiated small round cell sarcomas with BCOR gene mutations. The classification points to the importance of molecular diagnosis in the diagnosis and management of these rare tumors [7].

BCOR-CCNB3 sarcomas arise most commonly in bone with a weak predominance over soft tissue. The most common sites are the pelvis, lower extremities (e.g., femur and tibia), and paraspinal region [8]. Our 11-year-old female patient's femoral BCOR-CCNB3 tumor, measuring 6.4 × 5.5 × 4.0 cm, aligns with the typical age and tumor location for this type of cancer. However, her female sex is uncommon, as cohorts typically show about seven males for every female. Additionally, her tumor was smaller (6.4 cm) than average, which is usually around 11.7 cm [4].

BCOR-CCNB3 sarcoma is a rare, high-grade, undifferentiated small round cell sarcoma with a specific gene fusion between the BCOR (BCL6 corepressor) and CCNB3 (Cyclin B3) genes. The gene fusion is due to a paracentric inversion of the X chromosome and results in overexpression of BCOR, with a role in oncogenesis. Further research has confirmed that the BCOR-CCNB3 fusion leads to the overexpression of BCOR, contributing to tumor development. This overexpression is believed to drive cell proliferation, underscoring the oncogenic potential of the fusion [9]. CCNB3 encodes the testis-specific cyclin B gene, functions in mitosis, and connects CDK2 for spermatic production [10]. The precise function of BCOR::CCNB3 is still under investigation. However, when it was detected in NIH3T3 cells, high expression of the hedgehog or Wnt pathways was noted [6]. BCOR-CCNB3 sarcoma is predominantly seen in children and adolescents, with over 90% of the patients coming in under the age of 20. The average age at presentation is around 13.8 years. It has a high male predominance with a male-to-female ratio of approximately 6.7:1 [11]

This sarcoma arises most commonly in bone, with little predilection for soft tissue involvement (approximately 1.5:1). The most common anatomical sites to be involved are the long bones of the extremities, particularly the femur, tibia, and fibula, and the pelvis and paraspinal regions. The sites are identical to those of Ewing sarcoma [12]. Five-year OS of BCOR::CCNB3 sarcoma is approximately 72% to 75%, which is comparable to Ewing sarcoma and significantly better than CIC-rearranged sarcomas, which have a five-year OS of approximately 43% [4]. These outcomes are generally in the context of patients receiving multimodal treatment, including surgery and chemotherapy protocols that are the same or very similar to Ewing sarcoma. Histologically, BCOR CCNB3 sarcoma occurs typically as sheets or fascicles of spindle-to-round cells with monomorphic nuclei, subtle chromatin, and minimal cytoplasm. The stroma varies from myxoid to collagenous, sometimes with a concomitant fine capillary network or hemangiopericytic vascular pattern [4]. Mitosis varies from five to 60 per 10 high-power fields, reflecting its aggressive nature. Our case showed most of these depicting features.

In the immunohistochemical evaluation of BCOR-CCNB3 sarcoma, several markers play a critical role in differential diagnosis. CD56 is often positive, reflecting potential neural differentiation, though it lacks specificity as it is also expressed in Ewing sarcoma, neuroblastoma, and CIC-rearranged sarcomas. BCL2, a marker of anti-apoptotic signaling, is frequently positive in BCOR-CCNB3 sarcomas but is also seen in a wide range of other small round cell tumors, including synovial sarcoma and desmoplastic small round cell tumor (DSRCT). CD99, a commonly used marker in the diagnosis of round cell tumors, shows weak and patchy staining in BCOR-CCNB3 sarcomas, distinguishing it from Ewing sarcoma, where it exhibits strong, diffuse membranous positivity. This unique combination of immunohistochemical features, along with molecular studies for BCOR and CCNB3, formed the cornerstone of BCOR-CCNB3 sarcoma diagnosis in our case. Our differentials were ruled out due to the negative immunohistochemistry of NKX2.2, WT1, S-100, HMB-45, Desmin, and PanCK that ruled out Ewing sarcoma, CIC-DUX4 sarcoma, clear cell sarcoma of soft tissue, rhabdomyosarcoma, and synovial sarcoma, respectively [13].

The patient was on three courses of alternative chemotherapy using VDC/IE, based on the JESS04 protocol [14], which brought a marked improvement in the lesion size. The tumor responded favorably to the chemotherapy based on a protocol for Ewing sarcoma. A similar result of Ewing sarcoma-oriented chemotherapy for BCOR::CCNB3 sarcoma was already reported and was concordant with the published practice [4].

A striking feature of our case is the long symptomatic course, almost two years, which is decidedly longer than that usually seen in Ewing sarcoma. A rigorous literature review, however, failed to demonstrate any evident consensus regarding whether this prolonged symptomatic period can be a common clinical presentation of BCOR::CCNB3 sarcoma, underlining the necessity for further studies.

This case, while notable for the patient's young age and female sex, both less common for BCOR-CCNB3 sarcoma, is otherwise a typical presentation. The tumor's location in a long bone of a child, combined with treatment using an Ewing-style therapy, aligns well with established profiles for this disease. Therefore, the patient's prognosis is expected to be similar to the 75% five-year survival rate reported in the literature [15].

## Conclusions

We reported a rare case of BCOR::CCNB3 sarcoma in a child's femur initially with osteosarcoma-like clinical and radiologic presentation. This case underscores the value of BCOR-rearranged sarcomas to be included in the differential diagnosis of round-cell soft tissue and bone tumors in children. Accurate diagnosis can only be made by a combination of histopathology, immunohistochemistry, and, most critically, molecular analysis to detect typical gene fusions such as BCOR::CCNB3. Early molecular identification is crucial for an accurate diagnosis. It also helps in choosing the right therapy since BCOR-CCNB3 sarcomas have responded well to Ewing sarcoma chemotherapy. Additionally, molecular characterization can provide prognostic insights by differentiating this type from other Ewing-like sarcomas that have worse outcomes. Doctors should stay alert and carry out targeted molecular testing in cases where the histologic and immunophenotypic features are unclear. This approach can prevent misdiagnoses and improve patient management.

Notable characteristics of this case were an almost two-year asymptomatic period and the specific imaging feature of an inhomogeneous appearance on MRI. The diversity of clinical manifestations and imaging characteristics of BCOR::CCNB3 sarcoma is still not well known and should be further discussed in future reports to improve the management of similar cases.

## References

[1] Tan LC, Yu PC, Wang J, Zhou XY, Ji QH, Wang YL (2021). BCOR-CCNB3 rearranged sarcoma arising in neck misdiagnosed as thyroid cancer: a case report. Oral Oncol.

[2] Cloutier JM, Charville GW (2019). Diagnostic classification of soft tissue malignancies: a review and update from a surgical pathology perspective. Curr Probl Cancer.

[3] Specht K, Zhang L, Sung YS (2016). Novel BCOR-MAML3 and ZC3H7B-BCOR gene fusions in undifferentiated small blue round cell sarcomas. Am J Surg Pathol.

[4] Kao YC, Owosho AA, Sung YS (2018). BCOR-CCNB3 fusion positive sarcomas: a clinicopathologic and molecular analysis of 36 cases with comparison to morphologic spectrum and clinical behavior of other round cell sarcomas. Am J Surg Pathol.

[5] Dehner CA, Lazar AJ, Chrisinger JS (2024). Updates on WHO classification for small round cell tumors: Ewing sarcoma vs. everything else. Hum Pathol.

[6] Pierron G, Tirode F, Lucchesi C (2012). A new subtype of bone sarcoma defined by BCOR-CCNB3 gene fusion. Nat Genet.

[7] Sbaraglia M, Bellan E, Dei Tos AP (2021). The 2020 WHO Classification of Soft Tissue Tumours: news and perspectives. Pathologica.

[8] Bharucha P, Harvilla N, White R (2019). Unusual distal tibia BCOR sarcoma: a case report and review of imaging features. OAJ Clin Case Rep.

[9] Peters TL, Kumar V, Polikepahad S (2015). BCOR-CCNB3 fusions are frequent in undifferentiated sarcomas of male children. Mod Pathol.

[10] Nguyen TB, Manova K, Capodieci P (2002). Characterization and expression of mammalian cyclin b3, a prepachytene meiotic cyclin. J Biol Chem.

[11] Suzuki K, Yasuda T, Haruhara Y, Watanabe K, Nomura K, Kanamori M, Kawaguchi Y (2022). BCOR-CCNB3 sarcoma arising in the proximal tibia: a case report. Mol Clin Oncol.

[12] Kedia A, Singh G, Parmar S (2023). A rare case of lower limb sarcoma with BCOR-CCNB3 mutation: diagnosis and treatment. Cureus.

[13] Yoshida A, Sekine S, Tsuta K, Fukayama M, Furuta K, Tsuda H (2012). NKX2.2 is a useful immunohistochemical marker for Ewing sarcoma. Am J Surg Pathol.

[14] Chin M, Yokoyama R, Sumi M (2020). Multimodal treatment including standard chemotherapy with vincristine, doxorubicin, cyclophosphamide, ifosfamide, and etoposide for the Ewing sarcoma family of tumors in Japan: results of the Japan Ewing Sarcoma Study 04. Pediatr Blood Cancer.

[15] Imanishi J, Sato K, Kikuchi Y (2025). Update on the management of BCOR::CCNB3 sarcoma. Jpn J Clin Oncol.

